# *In vitro* antimicrobial efficacy of laser-synthesized silver nanoparticles against antibiotic-resistant *Escherichia coli* isolated from dairy cattle wastewater

**DOI:** 10.14202/vetworld.2026.511-522

**Published:** 2026-02-10

**Authors:** Sheila Marty Yanestria, Freshinta Jellia Wibisono, Mustofa Helmi Effendi, Tri Untari, Aswin Rafif Khairullah, Fidi Nur Aini Eka Puji Dameanti, John Yew Huat Tang, Saifur Rehman, Wasito Wasito, Riza Zainuddin Ahmad

**Affiliations:** 1 Department of Veterinary Public Health, Faculty of Veterinary Medicine, Universitas Wijaya Kusuma Surabaya, Jl. Dukuh Kupang XXV No.54, Dukuh Kupang, Dukuh Pakis, Surabaya, 60225, East Java, Indonesia; 2 Department of Veterinary Public Health, Faculty of Veterinary Medicine, Universitas Airlangga, Jl. Dr. Ir. H. Soekarno, Kampus C Mulyorejo, Surabaya 60115, East Java, Indonesia; 3 Research Group of Antimicrobial Resistance, Faculty of Veterinary Medicine, Universitas Airlangga Surabaya, 60115, East Java, Indonesia; 4 School of Food Industry, Faculty of Bioresources, and Food Industry, Universiti Sultan Zainal Abidin (Besut Campus), Besut 22200, Malaysia; 5 Department of Microbiology, Faculty of Veterinary Medicine, Universitas Gadjah Mada, Bulaksumur, Caturtunggal, Depok, Sleman 55281, Daerah Istimewa Yogyakarta, Indonesia; 6 Research Center for Veterinary Science, National Research and Innovation Agency (BRIN), Jl. Raya Bogor Km. 46 Cibinong, Bogor 16911, West Java, Indonesia; 7 Laboratory of Veterinary Microbiology and Immunology, Faculty of Veterinary Medicine, Universitas Brawijaya, Jl. Puncak Dieng, Kunci, Kalisongo, Dau, Malang 65151, East Java, Indonesia; 8 Department of Pathobiology, Faculty of Veterinary and Animal Sciences, Gomal University, RV9W+GVJ, Indus HWY, Dera Ismail Khan 27000, Pakistan

**Keywords:** antimicrobial resistance, dairy farm wastewater, *Escherichia coli*, laser ablation, One Health, silver nanoparticles, wastewater management, zoonotic bacteria

## Abstract

**Background and Aim:**

Poorly managed dairy farm wastewater is a significant reservoir of antibiotic-resistant bacteria, particularly *Escherichia coli*, contributing to the environmental spread of antimicrobial resistance (AMR) and posing risks to animal and public health. Conventional wastewater treatment systems are often insufficient to inactivate these resistant organisms. Silver nanoparticles (AgNPs), especially those synthesized by pulsed laser ablation (PLA) in liquid, offer a high-purity, chemical-free nanomaterial with promising antimicrobial properties. This study aimed to evaluate the *in vitro* antimicrobial efficacy of laser-synthesized AgNPs against antibiotic-resistant *E. coli* isolated from dairy cattle wastewater within a One Health framework.

**Materials and Methods:**

Wastewater samples were collected aseptically from 50 smallholder dairy farms in East Java, Indonesia. *E. coli* isolates were identified using standard cultural, morphological, Gram staining, and biochemical (Indole, methyl red, Voges–Proskauer, citrate) methods. Antibiotic resistance was screened using the Kirby–Bauer disk diffusion method against streptomycin, erythromycin, penicillin, and tetracycline. AgNPs were synthesized via PLA in polyvinylpyrrolidone medium and characterized using transmission electron microscopy, ultraviolet–visible spectroscopy, and Fourier transform infrared spectroscopy. The minimum inhibitory concentration (MIC) and minimum bactericidal concentration (MBC) of AgNPs were determined by broth microdilution and agar subculture methods, respectively, across concentrations ranging from 0.195 to 100 ppm. Statistical analysis was performed using one-way analysis of variance followed by Tukey’s post hoc test at a significance level of p < 0.05.

**Results:**

PLA successfully produced monodisperse AgNPs with a mean diameter of 11.62 ± 1.8 nm and a characteristic surface plasmon resonance peak at 418 nm, confirming high-purity and stability. Twenty antibiotic-resistant *E. coli* isolates were evaluated. MIC values ranged from 37.5 to 100 ppm, with erythromycin-resistant isolates showing the lowest MICs (45.0 ± 10.5 ppm) and streptomycin-resistant isolates the highest (75.0 ± 33.3 ppm). Most isolates (75%) exhibited MBC values >100 ppm, indicating predominantly bacteriostatic activity at the tested concentrations. No statistically significant differences in MIC values were observed among resistance groups (p > 0.05). A concentration of 62.5 ppm was identified as the most effective inhibitory dose across resistance profiles.

**Conclusion:**

Laser-synthesized AgNPs demonstrated consistent *in vitro* inhibitory activity against antibiotic-resistant *E. coli* from dairy wastewater, with an optimal MIC of approximately 62.5 ppm. These findings highlight the potential application of AgNPs as a supplementary control strategy in dairy waste management and AMR mitigation, supporting an integrated One Health approach.

## INTRODUCTION

Antimicrobials are a cornerstone of modern public health; however, the increasing prevalence of antimicrobial resistance (AMR), particularly in the livestock sector, poses a serious threat to the effectiveness of infectious disease therapy in both animals and humans [1, 2]. Poorly managed dairy farm waste represents a major source for the dissemination of antibiotic-resistant bacteria (ARB) [3]. Such waste frequently contains high concentrations of *Escherichia coli* harboring antibiotic resistance genes associated with antimicrobial use in veterinary production systems [4]. *E. coli* isolated from dairy cow waste has been shown to exhibit resistance to multiple antibiotic classes, including tetracyclines, beta-lactams, macrolides, and aminoglycosides [5, 6]. The presence of ARB in agricultural waste is not only indicative of on-farm management challenges but also constitutes a significant environmental and public health risk [7].

Conventional wastewater treatment systems are frequently inadequate for the effective inactivation of ARB [8], highlighting the urgent need for alternative control strategies. Silver nanoparticles (AgNPs) have demonstrated broad-spectrum antibacterial activity against a wide range of pathogenic bacteria, including antibiotic-resistant strains, under both *in vitro* and *in vivo* conditions [9, 10]. The antimicrobial activity of AgNPs is mediated by multiple nonspecific mechanisms, including disruption of bacterial cell membranes, induction of oxidative stress, and interference with essential intracellular biochemical processes, thereby limiting bacteria’s ability to develop resistance [11, 12]. The inhibitory and bactericidal effects of AgNPs against *E. coli* have been consistently reported [13–15].

In the present study, AgNPs were synthesized using pulsed laser ablation (PLA) in liquid, a technique that offers distinct advantages over conventional chemical synthesis approaches. PLA enables the production of AgNPs with very high-purity (>99%) without residual chemical contaminants, such as reducing agents, stabilizers, or capping agents, which are commonly associated with chemical synthesis and may interfere with antimicrobial assessment [16]. In addition, PLA-derived AgNPs exhibit uniform particle size, high surface reactivity, sustainability, and long-term colloidal stability. Consequently, PLA was selected as the most appropriate method to generate AgNPs with optimal characteristics for reliable antimicrobial evaluation against antibiotic-resistant *E. coli* isolates originating from livestock waste [17].

The minimum inhibitory concentration (MIC) and minimum bactericidal concentration (MBC) are critical parameters for determining effective antimicrobial doses and are widely used to assess the *in vitro* efficacy of AgNPs against pathogenic isolates from livestock waste, particularly *E. coli* [10]. Continued refinement of AgNP synthesis methods and evaluation at biologically relevant concentrations is expected to contribute to novel strategies for controlling ARB in the livestock sector and reducing the environmental and public health risks associated with AMR dissemination [9, 18].

Despite growing evidence supporting the antimicrobial potential of AgNPs against ARB, several critical gaps remain in the context of livestock-associated environmental contamination. Most existing studies rely on chemically synthesized or biologically derived AgNPs, which may contain residual reagents that confound antimicrobial assessment and limit reproducibility. In addition, many investigations use reference strains rather than field-derived ARB, reducing real-world relevance. Data on the *in vitro* efficacy of high-purity, PLA-synthesized AgNPs against antibiotic-resistant *E. coli* originating specifically from dairy farm wastewater are scarce. Moreover, comparative evaluations of MIC and MBC across different antibiotic resistance profiles of *E. coli* isolates remain limited, hindering the identification of an optimal inhibitory concentration applicable to heterogeneous resistance patterns. This lack of integrated evidence impedes the translation of AgNP-based interventions into practical livestock waste management strategies to mitigate AMR dissemination within a One Health framework.

Therefore, this study aimed to evaluate the *in vitro* antimicrobial efficacy of PLA-synthesized AgNPs against antibiotic-resistant *E. coli* isolated from dairy farm wastewater. Specifically, the study sought to determine and compare the MIC and MBC values of AgNPs across *E. coli* isolates exhibiting resistance to different antibiotic classes, and to identify an effective inhibitory concentration applicable across resistance profiles. By using high-purity, chemically uncontaminated AgNPs and field-derived isolates, this work aims to provide robust evidence supporting the potential application of AgNPs as a complementary control strategy for ARB in dairy waste systems and to contribute to AMR mitigation efforts under a One Health approach.

## MATERIALS AND METHODS

### Ethical approval

This study was approved by the Ethical Clearance Committee of the Faculty of Veterinary Medicine, Universitas Wijaya Kusuma Surabaya, Indonesia (Ethics number: 97-KKE/2025). All procedures were conducted in accordance with institutional ethical guidelines for microbiological research and environmental sampling. The collection, transportation, and handling of dairy farm wastewater samples followed established institutional biosafety protocols and relevant national regulations in Indonesia.

### Study period and location

The study was conducted from June to August 2025 at the Veterinary Public Health Laboratory, Faculty of Veterinary Medicine, Universitas Wijaya Kusuma Surabaya, Indonesia.

### Research design

A cross-sectional study was conducted to evaluate the antimicrobial effectiveness of AgNPs synthesized using PLA against antibiotic-resistant *E. coli* isolates obtained from dairy cow waste in the livestock area of Grati District, Pasuruan Regency, East Java.

### Sample collection

Wastewater samples (5 mL per farm) were collected from 50 smallholder dairy farms housing approximately 2–10 dairy cows each. Samples were obtained directly from drainage channels or wastewater storage tanks using sterile pipettes. To minimize external contamination, sampling was performed at a depth of 5–10 cm below the wastewater surface under aseptic conditions. Each sample was labeled with farm identity, date, time, and environmental conditions. All samples (n = 50) were transported to the laboratory in insulated containers with ice packs maintained at 4°C and processed within 2 h of collection.

### Synthesis of AgNPs using PLA

AgNPs were synthesized from silver metal plates, 5 × 10 × 20 mm³, 99.9% purity (Sigma-Aldrich, St. Louis, MO, USA), using polyvinylpyrrolidone (PVP) (Merck KGaA, Darmstadt, Germany) as the liquid medium. An Nd:YAG laser, 1064 nm wavelength, 7 ns pulse width, 20 Hz (Polaris II, New Wave Research, Fremont, CA, USA) was used as the radiation source, with laser parameters controlled using LaserExec II software (New Wave Research, Fremont, CA, USA). The laser energy was set at 30 mJ with a repetition rate of 10 Hz. Characterization was performed using a ultraviolet–visible (UV–Vis) spectrophotometer (Shimadzu Corporation, Kyoto, Japan), transmission electron microscopy (TEM; JEOL Ltd, Tokyo, Japan) coupled with energy-dispersive X-ray spectro-scopy, and Fourier transform infrared spectroscopy (FTIR) (Shimadzu Corporation, Kyoto, Japan) [19].

During synthesis, the laser beam was directed by a silver mirror and focused through a quartz lens (30 mm focal length) onto the silver target immersed in a liquid medium in a Petri dish for 11 h. The solution color gradually changed from transparent to light yellow and then to brownish yellow with increasing laser exposure. TEM, particle size analysis, UV–Vis spectrophotometer, and FTIR spectroscopy were used to assess morphology, particle size distribution, optical plasmon resonance, and surface chemistry [19]. The AgNP suspension was stored in sealed amber glass bottles at 4°C–8°C to maintain stability and prevent aggregation. Only a single batch was used in this study. The freshly synthesized AgNP colloid was diluted in sterile 0.9% NaCl to obtain an initial stock concentration of 1000 ppm.

### Isolation and identification of *E. coli*

Wastewater samples (1 mL) were inoculated into 9 mL of buffered peptone water (Himedia, Mumbai, India) and incubated at 37°C for 18–24 h for pre-enrichment. Subsequently, 0.1 mL of enriched culture was streaked onto MacConkey agar (Oxoid, Basingstoke, UK) and incubated at 37°C for 18–24 h under aerobic conditions. Lactose-fermenting colonies exhibiting pink to red coloration, smooth surface, round shape, and convex edges were selected for further analysis. Presumptive *E. coli* isolates were confirmed using morphological examination, Gram staining, and standard biochemical tests (Indole, methyl red, Voges–Proskauer, citrate) [20, 21].

### Antimicrobial susceptibility testing (AST)

Confirmed *E. coli* isolates were subjected to AST using the Kirby–Bauer disk diffusion method in accordance with Clinical and Laboratory Standards Institute (CLSI) M100, 34th edition standards [22]. Four antibiotic disks (Oxoid) were used for screening: penicillin (10 IU/disk), streptomycin (10 µg/disk), erythromycin (15 µg/disk), and tetracycline (30 µg/disk). Resistance breakpoints were defined as ≤11 mm for streptomycin and tetracycline, and ≤13 mm for penicillin, according to the CLSI 2024 guidelines [22]. As *E. coli* is not routinely tested for erythromycin under CLSI standards, a general resistance cutoff of ≤13 mm was applied. A total of 20 antibiotic-resistant *E. coli* isolates (five per antibiotic category) were selected for MIC and MBC assays [22, 23]. *E. coli* ATCC 25922 (Oxoid) was used as the quality control strain.

### Determination of MIC

Bacterial inocula were prepared by culturing selected *E. coli* isolates in Mueller–Hinton broth (MHB) (Oxoid, Basingstoke, UK) at 37°C for 18 h with agitation at 150 rpm until an OD_600_ of 0.5–0.6 McFarland standard was achieved, corresponding to approximately 1.5 × 10^8^ CFU/mL. MIC determination was performed using the broth microdilution method according to CLSI M07-A8 guidelines [24]. Serial twofold dilutions of AgNPs (100–0.195 ppm) were prepared in 96-well microplates (OneMed, Sidoarjo, Indonesia) containing MHB inoculated with standardized bacterial suspensions (10^8^ CFU/mL). Plates were incubated at 37°C for 24 h. The MIC endpoint was defined as the lowest AgNP concentration showing no visible bacterial growth, as assessed by visual turbidity before and after incubation. Negative and positive controls were included, and all assays were performed in duplicate.

### Determination of MBC

MBC determination was conducted using the conventional agar diffusion method. Aliquots (10 µL) from MIC wells without visible growth were spread onto Mueller–Hinton agar (Himedia) plates and incubated at 37°C for 24 h. The MBC endpoint was defined as the lowest AgNP concentration resulting in the complete absence of bacterial growth on agar plates, indicating total bacterial killing [25]. All tests were performed in duplicate.

### Statistical analysis

MIC and MBC values were analyzed using one-way analysis of variance followed by Tukey’s multiple comparison test to identify differences among antibiotic resistance groups. Statistical significance was set at p < 0.05. All analyses were performed using IBM SPSS Statistics version 29 (IBM Corp., Armonk, NY, USA).

## RESULTS

### Characterization of AgNPs

AgNPs were successfully synthesized using PLA in PVP media and demonstrated excellent physicochemical characteristics. TEM analysis of 247 particles showed an average particle diameter of 11.62 ± 1.8 nm, with a coefficient of variation of 15.5%, indicating a narrow and monodisperse size distribution. UV–Vis spectrophoto-meter revealed a distinct surface plasmon resonance peak at 418 nm with an absorbance intensity of 0.786 AU, confirming the formation of metallic silver without detectable silver oxide contamination. FTIR analysis verified the presence of PVP K30 as a capping and stabilizing agent on the AgNP surface, characterized by a strong peak at 1680 cm^-1^ (C = O stretching) and additional peaks at 2950, 1550, and 1250 cm^-m^, indicating an intact PVP backbone without degradation. The estimated coating thickness of approximately 2.6 nm contributed to colloidal stability. Overall, the synthesized AgNPs exhibited high purity, uniform morphology, and stability, making them suitable for subsequent antimicrobial evaluation (MIC/MBC) against antibiotic-resistant *E. coli* isolates from dairy cow waste.

### Antimicrobial activity against antibiotic-resistant *E. coli*

The antimicrobial activity of AgNPs was evaluated against antibiotic-resistant *E. coli* isolates recovered from dairy cattle waste. The isolates comprised streptomycin-resistant (S1–S5), erythromycin-resistant (E1–E5), penicillin-resistant (P1–P5), and tetracycline-resistant (T1–T5) groups.

**Figure 1:**
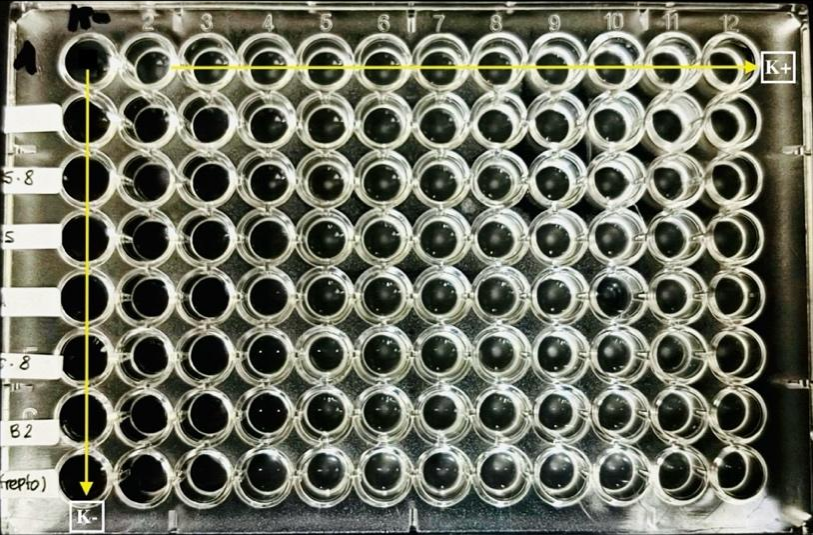
Results of the minimum inhibitory concen-tration assay of silver nanoparticles against antibiotic-resistant *Escherichia coli* were determined using the standard broth microdilution method. K− indicates the negative control with no bacterial growth (clear wells), whereas K+ indicates the positive control with bacterial growth (turbid wells).

**Figure 2:**
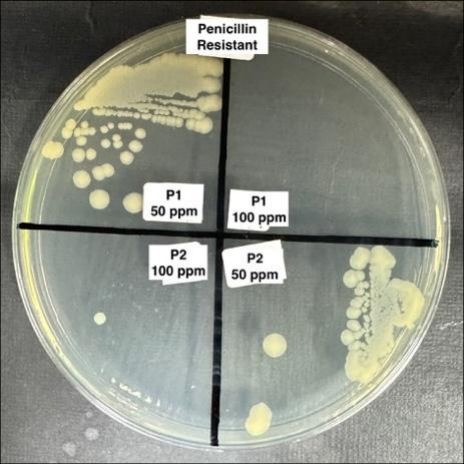
Results of the minimum bactericidal concentration assay of silver nanoparticles against antibiotic-resistant *Escherichia coli* determined using the conventional agar diffusion method on Mueller–Hinton agar. P denotes penicillin-resistant isolates.

Figure 1 presents the MIC results determined using the standard broth microdilution method. Following MIC determination, MBC assessment was performed by subculturing wells without visible growth onto Mueller–Hinton agar. As illustrated in Figure 2, AgNPs exerted a bactericidal effect at 100 ppm against isolate P1, whereas isolate P2 continued to grow at the same concentration.

Results of the minimum inhibitory concen-tration assay of silver nanoparticles against antibiotic-resistant *Escherichia coli* were determined using the standard broth microdilution method. K− indicates the negative control with no bacterial growth (clear wells), whereas K+ indicates the positive control with bacterial growth (turbid wells).

Results of the minimum bactericidal concentration assay of silver nanoparticles against antibiotic-resistant *Escherichia coli* determined using the conventional agar diffusion method on Mueller–Hinton agar. P denotes penicillin-resistant isolates.

### MIC and MBC distribution among resistance groups

The MIC and MBC values for all antibiotic-resistant *E. coli* isolates tested against AgNPs are summarized in Table 1. MIC values ranged from 37.5 to 100 ppm across all isolates. In contrast, MBC values were generally higher, with most isolates exhibiting MBC values exceeding 100 ppm, the highest concentration tested in this study. These findings indicate that AgNPs exerted a strong inhibitory (bacteriostatic) effect but comparatively weaker bactericidal activity against antibiotic-resistant *E. coli* isolated from dairy cattle waste.

**Table 1:** MIC and MBC of AgNPs against antibiotic-resistant *Escherichia coli* isolates recovered from dairy cattle wastewater.

Antibiotic	Isolate	Inhibition zone (mm)	CLSI resistance zone (mm)	MIC (ppm)	MBC (ppm)
Streptomycin	S1	11	≤11	75	>100
	S2	8		75	>100
	S3	10		62.5	>100
	S4	0		100	>100
	S5	8		62.5	>100
Erythromycin	E1	8	≤13	50	100
	E2	10		50	100
	E3	10		37.5	>100
	E4	10		50	100
	E5	10		37.5	>100
Penicillin	P1	0	≤13	50	100
	P2	0		37.5	>100
	P3	0		50	100
	P4	0		62.5	>100
	P5	0		62.5	>100
Tetracycline	T1	0	≤11	62.5	>100
	T2	0		75	>100
	T3	0		37.5	>100
	T4	0		50	>100
	T5	8		62.5	>100

AgNPs = Silver nanoparticles, MIC = Minimum inhibitory concentration, MBC = Minimum bactericidal concentration, CLSI = Clinical and Laboratory Standards Institute. Values >100 ppm indicate no bactericidal activity at the highest concentration tested.

After 24 h of incubation, streptomycin-resistant *E. coli* required the highest AgNP concentrations for growth inhibition, with MIC values ranging from 62.5 to 100 ppm. In contrast, erythromycin-resistant *E. coli* showed the lowest MIC values among the resistance groups, ranging from 37.5 to 50 ppm. Across all isolates, MBC values exceeded corresponding MIC values and ranged between 100 and >100 ppm. Overall, 75% (15/20) of the isolates exhibited MBC values >100 ppm. Only five isolates showed an MBC of 100 ppm, comprising three erythromycin-resistant and two penicillin-resistant *E. coli* isolates. Due to the predominance of MBC values >100 ppm, further MBC interpretation was limited to descriptive analysis.

### Comparative statistical analysis of MIC values

One-way ANOVA followed by Tukey’s multiple comparison test revealed no statistically significant differences in mean MIC values among the antibiotic resistance groups (F (3,36) = 2.20; p = 0.105). Post hoc Tukey’s Honestly Significant Differences analysis confirmed that none of the pairwise comparisons reached statistical significance (p > 0.05). Descriptively, streptomycin-resistant isolates exhibited the highest mean MIC values (75.0 ± 33.3 ppm), whereas erythromycin-resistant isolates showed the lowest mean MIC values with the narrowest variability (45.0 ± 10.5 ppm). These data are presented in Table 2.

**Table 2:** Distribution of MBC of AgNPs across antibiotic resistance categories of *Escherichia coli* isolates recovered from dairy cattle wastewater.

Resistant category	n	Mean ± SD	Range
Streptomycin	10	75.0 ± 33.3	25–100
Erythromycin	10	45.0 ± 10.5	25–50
Penicillin	10	52.5 ± 27.5	25–100
Tetracycline	10	57.5 ± 31.3	25–100

AgNPs = Silver nanoparticles, MBC = Minimum bactericidal concentration, SD = Standard deviation. Values represent ppm.

### Identification of the most effective inhibitory concentration

**Figure 3:**
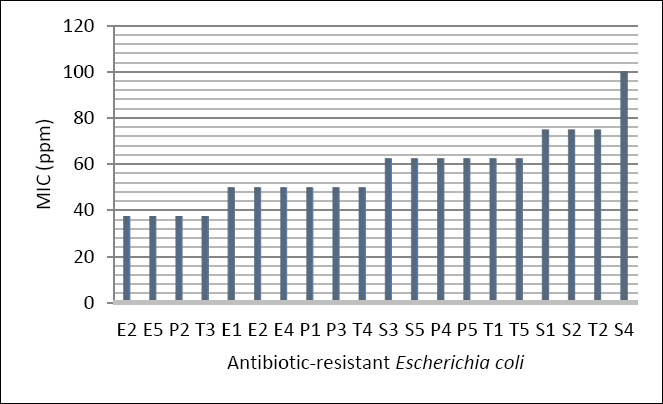
Minimum inhibitory concentration of silver nanoparticles against antibiotic-resistant *Escherichia coli*. Data bars represent the mean of duplicate measurements for each isolate. S = Streptomycin-resistant, E = Erythromycin-resistant, P = Penicillin-resistant, T = Tetracycline-resistant.

The lowest MIC value observed was 37.5 ppm, which inhibited erythromycin-resistant *E. coli* isolates (E2 and E5), one penicillin-resistant isolate (P2), and one tetracycline-resistant isolate (T3). At this concentration, streptomycin-resistant *E. coli* isolates were not inhibited (Figure 3). Growth inhibition of streptomycin-resistant isolates was first observed at an MIC of 62.5 ppm. Therefore, 62.5 ppm was identified as the most effective concentration for consistently inhibiting the growth of antibiotic-resistant *E. coli* isolated from dairy cattle waste across resistance profiles.

Minimum inhibitory concentration of silver nanoparticles against antibiotic-resistant *Escherichia coli*. Data bars represent the mean of duplicate measurements for each isolate. S = Streptomycin-resistant, E = Erythromycin-resistant, P = Penicillin-resistant, T = Tetracycline-resistant.

## DISCUSSION

### Antimicrobial efficacy of AgNPs against antibiotic-resistant *E. coli*

AgNPs have been extensively investigated as alternative antimicrobial agents against ARB due to their broad-spectrum activity. In the present study, the antimicrobial efficacy of AgNPs was evaluated against *E. coli* isolates resistant to streptomycin, erythromycin, penicillin, and tetracycline recovered from dairy cow waste. The observed MIC values ranged from 37.5 to 100 ppm, indicating effective growth inhibition within this concentration range. A serial twofold dilution assay (100–0.195 ppm) using a standardized bacterial inoculum of 10^8^ CFU/mL was applied, consistent with commonly adopted nanomaterial antimicrobial testing protocols, thereby ensuring data reliability and comparability with previous studies [26, 27].

### Variation in MIC among resistance phenotypes

The lowest MIC values were recorded for erythromycin-resistant *E. coli* (37.5–50 ppm), whereas streptomycin-resistant isolates required higher AgNP concentrations (62.5–100 ppm) for growth inhibition [28]. These differences likely reflect variability in resistance mechanisms, including efflux pump activity or antibiotic target modifications, which may also influence nanoparticle–cell interactions [27]. Streptomycin-resistant *E. coli* may possess enhanced protective mechanisms against metal nanoparticles, such as altered membrane permeability or more efficient efflux systems, reducing nanoparticle penetration and intracellular accumulation [29, 30].

### Statistical comparison of inhibitory effects

One-way ANOVA followed by Tukey’s multiple comparison test showed no statistically significant differences in MIC values among the antibiotic resistance groups, indicating that variations in AgNP concentration within the tested range did not significantly alter overall inhibitory efficacy. This suggests a relatively narrow effective concentration window for AgNP-mediated growth inhibition, within which concentration-dependent differences are not statistically distinguishable [31]. Streptomycin-resistant *E. coli* required an MIC of 62.5 ppm for inhibition, whereas erythromycin-, penicillin-, and tetracycline-resistant isolates were inhibited at concentrations below 62.5 ppm. Accordingly, 62.5 ppm was identified as the most effective concentration for consistent inhibition of antibiotic-resistant *E. coli* isolated from dairy cattle waste.

### Bacteriostatic versus bactericidal activity

MBC values exceeded MIC values for most isolates and frequently surpassed the maximum tested concentration of 100 ppm, indicating limited bactericidal activity under the conditions tested. These findings support the notion that AgNPs primarily exert a bacteriostatic effect against these isolates rather than direct bactericidal action [32]. Similar observations have been reported previously, where bacterial survival and metabolic activity persisted under sublethal AgNP exposure, suggesting that higher concentrations or combination strategies may be required to achieve complete bacterial killing [33]. The antimicrobial mechanisms of AgNPs, including membrane disruption, Reactive oxygen species (ROS) generation, and interactions with proteins and DNA, contribute to growth inhibition and, at higher doses, cell death [34].

### Influence of biofilm formation and heteroresistance

Bacterial biofilm formation and antibiotic resistance profiles play a critical role in determining sensitivity to AgNPs. Biofilm-producing bacteria exhibit increased resistance due to the extracellular matrix acting as a physical barrier that limits nanoparticle penetration, along with adaptive responses such as upregulation of efflux pump–related genes associated with metal resistance [35, 36]. These adaptations may promote cross-resistance to both antibiotics and AgNPs, highlighting the need for combination therapies or biofilm-targeted approaches [37, 38]. The substantial variability in MIC values observed within resistance groups can be attributed to multiple interacting factors, including heterogeneous resistance mechanisms, media-induced changes in AgNP stability and Ag^+^ ion availability [39], intrinsic heteroresistance within bacterial populations [40], and differences in biofilm-forming capacity among isolates [41].

### Mechanisms of action and adaptive responses

AgNPs exert antimicrobial activity through ROS generation, membrane damage, increased permeability, and interference with bacterial proteins and DNA. Because these mechanisms differ from the specific targets of conventional antibiotics, AgNPs generally remain effective against ARB and present a lower risk of resistance development [26, 42]. Nevertheless, adaptive resistance may emerge through mutations affecting efflux systems, membrane proteins, and stress response pathways following prolonged AgNP exposure [43, 44].

### Impact of nanoparticle aggregation on antimicrobial activity

The relatively high MIC values observed in this study may be partly explained by AgNP aggregation within the PVP matrix. Although PVP enhances colloidal stability, it may reduce Ag^+^ ion release and diminish antimicrobial efficacy. Under biorelevant conditions, AgNPs may aggregate to the micron scale, substantially reducing biological activity by decreasing surface area and particle–cell contact [45]. Previous studies have demonstrated that aggregation can alter MIC values by up to two orders of magnitude [46]. The MIC range observed here (37.5–100 ppm) is consistent with this phenomenon, as non-aggregated AgNPs of similar size typically exhibit MICs in the μg/mL range. Reduced Ag^+^ bioavailability, limited particle–bacteria interactions, and heterogeneous surface properties likely contributed to the predominance of MBC values >100 ppm and the identification of 62.5 ppm as the optimal inhibitory concentration [45–47].

### Comparative performance and formulation optimization

The effectiveness of AgNPs compared with conventional antibiotics highlights their potential as alternative antimicrobial agents against antibiotic-resistant *E. coli*. However, these findings also underscore the need for improved nanoparticle formulations or synergistic combinations with antibiotics to enhance bactericidal activity [28, 48]. Strategies to overcome aggregation include replacing PVP with alternative stabilizers such as dextran or plant-derived compounds, or applying surface coatings such as polydopamine or mesoporous silica to improve Ag^+^ release and antimicrobial performance [41, 49]. Such approaches have been shown to reduce MIC values to more competitive ranges (5–20 ppm) [39].

### Comparison with published studies

In this study, AgNPs effectively inhibited the growth of 20 antibiotic-resistant *E. coli* isolates from dairy farm waste, with MIC values ranging from 37.5 to 100 ppm. Erythromycin-resistant isolates exhibited the highest sensitivity, whereas streptomycin-resistant isolates required higher concentrations, reflecting heterogeneous resistance patterns. These MIC values are comparable to or lower than those reported in several published studies [50, 51]. For example, Trzcińska-Wencel *et al*. [50] reported MIC values of 16–64 μg/mL against *E. coli* ATCC with MBCs of 32–512 μg/mL, while Tufail *et al*. [51] reported MICs of 3.3–3.6 μg/mL for biogenic AgNPs. Rana *et al*. [52] reported an MIC of 1 mg/mL against *E. coli* MTCC 1698. The MIC values observed here (0.0375–0.1 mg/mL) are particularly noteworthy given that they were obtained using field-derived antibiotic-resistant isolates rather than more susceptible reference strains.

### Practical implications and One Health relevance

Evaluating *E. coli* isolates from dairy cow waste has direct relevance to veterinary medicine and livestock waste management. The application of antimicrobial agents such as AgNPs in waste treatment systems may reduce environmental dissemination of resistant bacteria and limit exposure risks to animals and humans. However, further research is required to optimize dosing strategies while considering long-term environmental safety and potential nanoparticle residues in animal products [53, 54].

This study aligns strongly with the One Health framework, which integrates human, animal, and environmental health. Dairy cattle waste represents a significant reservoir of ARB capable of transmission through multiple environmental pathways [55, 56]. Inadequately treated agricultural waste can contaminate soil, surface water, and drinking water sources, posing sustained public health risks [57, 58]. The demonstrated antimicrobial activity of AgNPs against antibiotic-resistant *E. coli* supports the role of nanotechnology in strengthening One Health–based AMR mitigation strategies, particularly by reducing pathogen spillover from livestock systems to the wider human population [59]. By identifying 62.5 ppm as an effective inhibitory concentration across resistance profiles, this study offers a scalable technological option for integration into modern livestock waste management and cross-sectoral AMR risk reduction programs [60].

## CONCLUSION

This study demonstrated that PLA-synthesized AgNPs effectively inhibited the growth of antibiotic-resistant *E. coli* isolated from dairy cattle waste. The MIC values ranged from 37.5 to 100 ppm, with erythromycin-resistant isolates showing the highest sensitivity (37.5–50 ppm) and streptomycin-resistant isolates requiring higher concentrations (62.5–100 ppm). Most isolates exhibited MBC values >100 ppm, indicating that AgNPs predominantly exerted a bacteriostatic rather than bactericidal effect under the tested conditions. Statistical analysis revealed no significant differences in MIC values among resistance groups (p > 0.05), and 62.5 ppm was identified as the most effective concentration for consistent growth inhibition across resistance profiles.

The observed inhibitory efficacy of AgNPs against antibiotic-resistant *E. coli* supports their potential application as a supplementary control measure in dairy waste management systems. Incorporation of AgNP-based treatments as a pre-treatment or polishing step in wastewater handling may reduce the environmental dissemination of ARB and lower exposure risks for livestock and surrounding communities. These findings are particularly relevant for resource-limited settings where conventional wastewater treatment methods are insufficient.

Key strengths include the use of high-purity, PLA-synthesized AgNPs free from chemical synthesis residues and the evaluation of field-derived antibiotic-resistant *E. coli* isolates rather than reference strains. The comprehensive assessment of MIC and MBC across multiple resistance phenotypes enhances the real-world relevance of the findings within a livestock and environmental health context.

This study was limited to *in vitro* testing under controlled laboratory conditions and did not assess AgNP performance in complex wastewater matrices or field-scale systems. The predominance of MBC values >100 ppm indicates limited bactericidal activity at the concentrations tested. In addition, potential environmental impacts, nanoparticle persistence, and toxicity to non-target organisms were not evaluated.

Future research should focus on optimizing AgNP formulations to enhance bactericidal efficacy, including surface modification, alternative stabilizers, or synergistic combinations with antibiotics or other antimicrobials. Evaluation of AgNP performance in real wastewater systems, assessment of long-term environmental safety, and investigation of resistance development under prolonged exposure are also warranted. Integration of AgNP-based interventions into holistic AMR mitigation strategies should be explored within a One Health framework.

Overall, PLA-synthesized AgNPs represent a promising non-antibiotic approach for inhibiting antibiotic-resistant *E. coli* in dairy cattle waste. By identifying 62.5 ppm as an effective inhibitory concentration across resistance profiles, this study provides a scientific basis for the potential integration of nanotechnology-based solutions into livestock waste management and AMR risk reduction strategies.

## DATA AVAILABILITY

All the generated data are included in the manuscript.

## AUTHORS’ CONTRIBUTIONS

SMY, MHE, and ARK: Conceptualization. WW, MHE, SMY, and RZA: methodology. JYHT, SR, and FJW: Formal analysis. TU, WW, and ARK: Investigation. JYHT, WW, RZA, and FJW: Data curation. TU and ARK: Visualization. FNAEPD, RZA, MHE, and WW: Validation. SMY, ARK, and MHE: Writing-original draft preparation. SMY, MHE, JYHT, FJW, TU, ARK, WW, RZA, FNAEPD, and SR: Writing-review and editing. SMY, ARK, MHE, and RZA: Supervision. All authors have read, critically reviewed, and approved the final manuscript, and agreed to be accountable for all aspects of the work.

## COMPETING INTERESTS

The authors declare that they have no competing interests.

## PUBLISHER’S NOTE

Veterinary World remains neutral with regard to jurisdictional claims in the published institutional affiliations.
